# Topical cenegermin associated ocular surface and contact lens drug precipitate deposit formation

**DOI:** 10.1016/j.ajoc.2022.101584

**Published:** 2022-05-14

**Authors:** Craig A. White, John Affeldt

**Affiliations:** Loma Linda University Eye Institute, 11370 Anderson St, Ste 1800, Loma Linda, CA, 92354, USA

**Keywords:** Cenegermin, Oxervate, Cornea, Deposits, Neurotrophic, Keratitis

## Abstract

**Purpose:**

To report for the first time drug precipitate deposit formation on both the ocular surface and bandage contact lens (BCL) of a patient treated with topical cenegermin.

**Observations:**

A patient suffering from stage III neurotrophic keratitis developed extensive ocular surface and BCL deposits over the eight week course of her topical cenegermin therapy. The ocular surface deposits were weakly adherent, detaching and clearing from the cornea within minutes of BCL removal. They reappeared rapidly and repeatedly however after each of five BCL exchanges. Symptom wise, the patient was unaware of their presence. Of historical note, this patient: (1) developed BCL ciprofloxacin deposits while undergoing a traditional neurotrophic keratitis treatment regimen, (2) did not develop corneal drug precipitate deposits within the placebo arm of the cenegermin clinical trial (vehicle only without cenegermin or BCL), and (3) did not develop corneal deposits with a latter BCL-free cenegermin treatment course.

**Conclusions and Importance:**

Topical cenegermin can produce extensive drug precipitate deposits on both the ocular surface and contact lens when used in conjunction with a bandage contact lens. Such deposits: (1) may represent an esthetic issue only as at least in our patient they were not symptom provoking, questionably interfered with the clinical course of the cenegermin therapy and did not require drug cessation, (2) may implicate both contact lenses and high frequency drug application as previously unidentified but formal risk factors for drug precipitate deposit formation, and (3) may act as a time-release medication reservoir enhancing drug delivery and long-term treatment efficacy.

## 1 Introduction

Cenegermin (Oxervate™, Dompé) is recombinant human nerve growth factor (hNGF) approved with orphan drug status and awarded *Breakthrough Therapy* designation by the FDA in 2018 for treatment of all stages of neurotrophic keratitis (NTK). As defined by the Mackie system^1^ and utilized in both the European and USA cenegermin phase II clinical trials,^2^^,^^3^ NTK classification includes stage I (punctate keratitis), stage II (persistent epithelial defect or PED), and stage III (corneal stromal melt). At various times, the patient presented in this report manifested all three of these NTK stages.

The molecular structure of cenegermin is identical to the hNGF produced in ocular tissues.^4^ Endogenous hNGF is believed to support corneal integrity through three mechanisms: corneal innervation, reflex tear secretion, and corneal epithelial cell proliferation and differentiation.^5^, ^6^, ^7^

Traditional therapies for NTK have included preservative-free topical lubrication, topical autologous serum, punctal occlusion, bandage contact lenses, amniotic membrane transplantation, tarsorrhaphy, and conjunctival flap. Cenegermin represents the first FDA-approved pharmacologic intervention for neurotrophic keratitis and addresses for the first time the root pathogenesis of the disease.

The most common drug-related adverse events identified in the cenegermin phase II clinical trials included eye pain, foreign body sensation, ocular inflammation, tearing, and corneal drug deposition (2/127 patients; 1.5%).^2^^,^^3^ In contradistinction and to the best of our knowledge, there have never been any reported cases of cenegermin associated ocular surface as well as bandage contact lens deposits.

Given that: (1) cenegermin represents a new, very promising, and increasingly utilized treatment for NTK, (2) contact lenses were prohibited in the cenegermin clinical trials, (3) drug application while wearing contact lenses (either corrective or therapeutic) is expressly prohibited under the “warnings and precautions” section of the package insert,^8^ (4) BCL's are frequently utilized for NTK in clinical practice, (5) the contact lenses in this case may have acted as a deposit forming promoter, and (6) the deposits may have enhanced the drug's clinical efficacy, the appearance of and potential treatment effects of cenegermin related corneal and/or contact lens deposits deserves documentation and discussion.

## 2 Case report

A 69-year-old Caucasian female presented in 2013 for care of her right eye corneal transplant. The transplant was necessitated by corneal scarring from previous herpes zoster ophthalmicus. She had developed symptomatic band keratopathy and in November 2014 underwent EDTA chelation. Postoperatively the patient developed a PED which proved refractory to traditional NTK therapy including aggressive topical lubrication, silicone plug and thermal punctal occlusion, serial BCL's, and prophylactic topical ciprofloxacin. During this treatment, she developed recurrent ciprofloxacin drug deposits on her BCLs.

In May 2015, the patient was enrolled in the USA phase II clinical trial of cenegermin.^3^ She was ultimately randomized to vehicle only, was protocol prohibited from wearing BCLs, did not develop corneal precipitate deposits, and was study discontinued after seven weeks secondary to ocular inflammation requiring topical steroid therapy. During study treatment, quantitated serial (4) corneal sensation measurements by Cochet-Bonnet esthesiometry repeatedly registered 0 mm in all four corneal quadrants. Her PED was not controlled until September 2015, some 10 months after the original EDTA chelation.

In January 2017 she underwent cataract extraction with intraocular lens implant of the right eye. Postoperatively she again developed intermittent PEDs, and her vision remained limited to 20/100 due to corneal graft scarring. In August 2019, with active stage I NTK and in preparation for repeat corneal transplantation, she underwent a full eight-week treatment course of topical cenegermin. During this period, she concomitantly received erythromycin ointment at bedtime, prednisolone acetate 1% about once weekly, did not wear BCLs, and did not develop corneal precipitate deposits.

In January 2020, some three months after completing cenegermin treatment, she underwent repeat penetrating keratoplasty of the right eye. Preoperative central corneal sensation measurements by Cochet-Bonnet esthesiometry remained unchanged from previous readings at 0 mm. Postoperatively she once again developed intermittent PEDs with associated mild stromal thinning (stage III NTK). Two months postoperatively she was started on a second eight-week course of topical cenegermin. Concomitant medications included moxifloxacin four times daily, difluprednate once daily (later exchanged for prednisolone acetate 1% once daily), erythromycin ointment at bedtime, and serial (5) BCL exchanges (Acuvue Oasys, plano sphere, BC 8.8, dia 14.0).

**Fig. 1:**
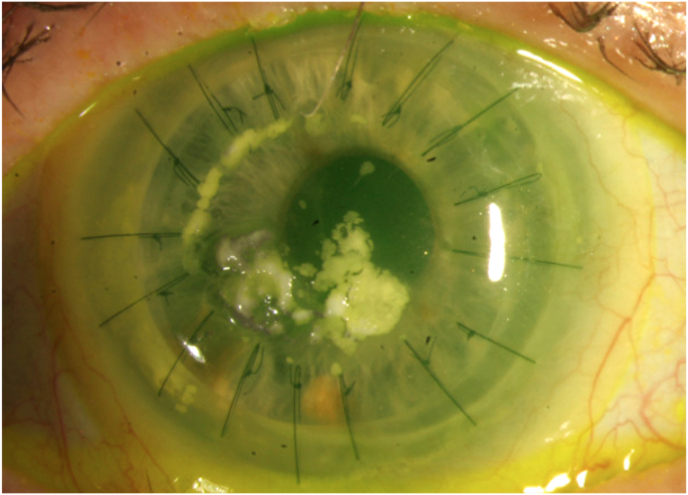
Slit lamp photograph of the right eye with bandage soft contact lens in place after instillation of fluorescein dye, demonstrating the initial white corneal deposits.

**Fig. 2:**
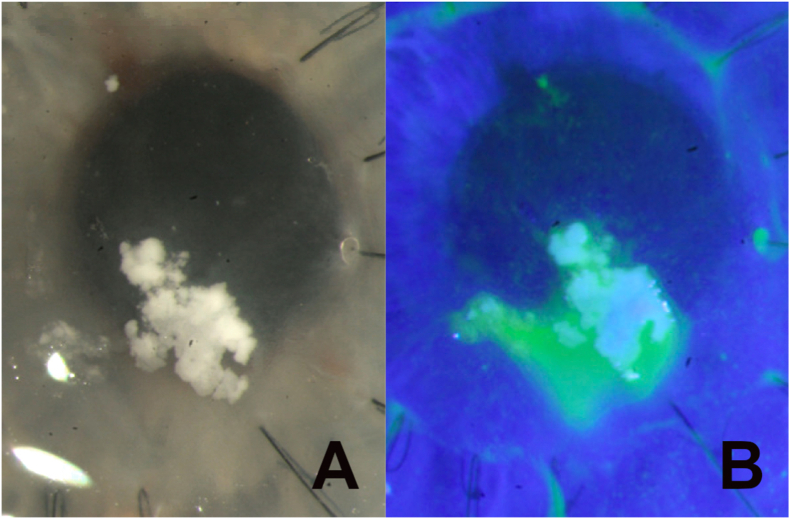
Slit lamp photograph of the right eye before (A) and after (B) instillation of fluorescein dye, demonstrating recurrence of white corneal deposits within the area of epithelial defect.

Four days after initiating cenegermin, the patient returned and was found to have extensive opaque white crystalline deposits on both her cornea and BCL (Fig. 1). The ocular surface deposits were located on both epithelialized and ulcerated cornea, but detached and cleared within minutes of contact lens removal. The BCL was replaced and all treatment continued. The patient returned 12 days later and was noted to have new ocular surface and BCL deposits (Fig. 2).

Recurrence of both corneal and BCL deposits was noted at each exam thereafter (6) during the eight-week cenegermin treatment course. They did so despite serial (5) BCL exchanges. The patient remained symptom-free and was unaware of deposit presence. Other than their disconcerting aesthetic appearance, the deposits did not appear to interfere with the cenegermin treatment course and did not require drug cessation. The PED resolved two months after completion of cenegermin therapy. By three months after treatment completion, central corneal sensation by Cochet-Bonnet esthesiometry had increased significantly to 20 mm. This increase was in sharp contrast to the first cenegermin intervention where three-month post-treatment central corneal sensation remained at 0 mm. 23 months after the second cenegermin intervention, corneal sensation had risen further to 45 mm with complete resolution of active NTK.

## 3 Discussion

Topical ophthalmic medication precipitates can be differentiated from systemic medication related corneal opacities (such as amiodarone, hydroxychloroquine, tamoxifen, and indomethacin) by their appearance, location, and configuration. The former present as white crystalline deposits on the corneal surface and/or contact lens, while the latter appear as intraepithelial opacities typically distributed in a verticillate or vortex keratopathy pattern.^9^

Topical medications reported to form precipitate ocular surface and/or contact lens deposits are surprisingly limited and include only the fluoroquinolone family of antibiotics. In historical order, they include 1st generation norfloxacin,^10^^,^^11^ 2nd generation pefloxacin,^12^ ofloxacin,^13^^,^^14^ and ciprofloxacin,^15^, ^16^, ^17^, ^18^ and 4th generation tosufloxacin.^19^ Additionally, there is a single report of corneal precipitate formation in a patient using moxifloxacin every 2 h for 38 days after corneal collagen cross linking.^20^ Clinically, fluoroquinolone precipitates tend to appear within the first few days of treatment,^14^^,^^16^^,^^17^^,^^19^ resolve within days of drug cessation,^16^ but can persist for weeks to months.^14^^,^^16^^,^^17^^,^^19^ In some cases, they have required surgical debridement.^17^ The patient had also been using preservative-free prednisolone acetate four times daily. A review of the literature did not reveal any reports of corneal deposits with the use of prednisolone.

A report by Qureshi et al. (2022) noted corneal deposits in five patients being treated with cenegermin for NTK.^21^ However, the deposits resembled band keratopathy, involved the corneal stroma rather than the corneal surface, did not resolve spontaneously with cessation of cenegermin, and, in one patient, were confirmed to consist of calcification of the corneal stroma on histopathologic examination.

Formally identified risk factors for corneal surface deposits include only multiple concomitant topical medications (polypharmacy)^14^ and older age.^16^ Literature review, however, additionally suggests that high frequency drug application represents a commonly associated but formally unrecognized deposit risk factor. Pooled data from precipitate reports which documented drug application frequency showed 104/109 cases (95%) on medication regimens ranging from 6 to 48 times per day.^11^^,^^14^^,^^16^^,^^17^^,^^22^^,^^23^

Although the cenegermin in our case may represent the first topical non-fluoroquinolone agent to produce ocular surface drug deposits, other than loose adherence, their clinical appearance and behavior were essentially identical to that of reported fluoroquinolone precipitates. The cenegermin deposits were whitish and crystalline in appearance, developed rapidly (within four days) after drug initiation, were part of a polypharmacy treatment protocol, developed in an older patient (age 69), and were associated with frequent drug application (six times daily).

It is instructive to note that: (1) during an earlier non-cenegermin NTK treatment course, the patient did develop ciprofloxacin BCL precipitates, (2) during her seven-week phase II cenegermin clinical trial where she received only vehicle without use of a BCL, she did not develop deposits, (3) during her first cenegermin treatment course where BCLs were not utilized, she did not develop corneal deposits, and (4) during her second cenegermin intervention where BCLs were utilized, she did develop both BCL and corneal deposits. This suggests that BCLs may have acted as a precipitate promotor. As such, contact lenses could represent a previously unrecognized but additional formal ocular surface drug precipitate deposit risk factor. Further research is needed to determine how differences in contact lens material and packaging solution might influence the propensity to promote medication deposition or to absorb medication from the tear film.

It is of additional interest that this patient developed precipitate deposits from both topical ciprofloxacin and cenegermin. This observation may reflect an individualized physiologic deposit propensity and renders her the first patient reported to suffer precipitates from two different classes of topical medication.

Finally, topical medication-associated corneal deposits can have a variety of effects. Adversely they can reduce vision, produce irritation and foreign body sensation, and slow epithelial cell migration and surface defect resolution.^16^^,^^17^^,^^22^ Advantageously, they may act as a medication depot. Quantified analysis of ciprofloxacin treatment associated deposits by high performance liquid chromatography and microbiologic assay not only validated the precipitates as ciprofloxacin but demonstrated them to possess high therapeutic levels of pharmacologic activity.^23^ This could result in a medication time-release effect, thereby enhancing drug delivery and therapeutic efficacy.

The potential problem of deposit delayed epithelial defect healing is of particular concern with cenegermin where therapeutic efficacy is primarily determined by defect closure. It is unclear in our case if the deposits slowed the healing process. Although ulcer resolution did not occur until two months after completion of cenegermin therapy, it is important to remember that despite a previous full eight-week course of cenegermin (where BCLs were not utilized and precipitates were not observed), the patient's three-month post treatment central corneal sensation measurements remained profoundly reduced and unchanged from pretreatment measurements of 0 mm. These measurements significantly improved three months after the second (and precipitate associated) cenegermin treatment course to 20 mm, and further increased to 45 mm by 23 months after treatment.

It is possible that this patient's ocular surface and/or BCL cenegermin deposits were treatment synergistic, acting as a drug reservoir to increase medication contact time, improve target tissue drug delivery, and ultimately enhance corneal reinnervation and NTK treatment success. If true, the addition of BCLs to a cenegermin treatment regimen with subsequent deposit formation could represent a desirable if not intentional therapeutic goal, as opposed to an adverse treatment event.

## 4 Conclusions

Cenegermin may be a second topical ophthalmic medication capable of producing deposits on the ocular surface and contact lens of treated patients, in addition to the well-recognized example of fluoroquinolone antibiotics. This propensity is of uncertain clinical significance, but may allow for improved treatment efficacy. Ultimately, it could challenge the theory-based manufacturer's admonition against the concurrent use of cenegermin and contact lenses.

## Patient consent

Consent to publish this case report has been obtained from the patient in writing.

## Funding

No funding or grant support.

## Authorship

All authors attest that they meet the current ICMJE criteria for authorship.

## Intellectual property

We confirm that we have given due consideration to the protection of intellectual property associated with this work and that there are no impediments to publication, including the timing of publication, with respect to intellectual property. In so doing we confirm that we have followed the regulations of our institutions concerning intellectual property.

## Research ethics

We further confirm that any aspect of the work covered in this manuscript that has involved human patients has been conducted with the ethical approval of all relevant bodies and that such approvals are acknowledged within the manuscript.

Written consent to publish potentially identifying information, such as details or the case and photographs, was obtained from the patient.

## Declaration of competing interest

The following authors have no financial disclosures: CW, JA.
